# Ca^2+^ Signaling in Cardiovascular Fibroblasts

**DOI:** 10.3390/biom14111365

**Published:** 2024-10-27

**Authors:** Andreas Rinne, Florentina Pluteanu

**Affiliations:** 1 Department of Biophysics and Cellular Biotechnology, University of Medicine and Pharmacy “Carol Davila” Bucharest, 050474 Bucharest, Romania; andreas.rinne@umfcd.ro; 2 Department of Anatomy, Animal Physiology and Biophysics, Faculty of Biology, University of Bucharest, 050095 Bucharest, Romania

**Keywords:** fibroblast, myofibroblast, Ca^2+^ signaling, cardiac fibrosis, pulmonary fibrosis, Ca^2+^ ion channels, Ca^2+^ transport mechanisms

## Abstract

Fibrogenesis is a physiological process required for wound healing and tissue repair. It is induced by activation of quiescent fibroblasts, which first proliferate and then change their phenotype into migratory, contractile myofibroblasts. Myofibroblasts secrete extracellular matrix proteins, such as collagen, to form a scar. Once the healing process is terminated, most myofibroblasts undergo apoptosis. However, in some tissues, such as the heart, myofibroblasts remain active and sensitive to neurohumoral factors and inflammatory mediators, which lead eventually to excessive organ fibrosis. Many cellular processes involved in fibroblast activation, including cell proliferation, protein secretion and cell contraction, are highly regulated by intracellular Ca^2+^ signals. This review summarizes current research on Ca^2+^ signaling pathways underlying fibroblast activation. We present receptor- and ion channel-mediated Ca^2+^ signaling pathways, discuss how localized Ca^2+^ signals of the cell nucleus may be involved in fibroblast activation and present Ca^2+^-sensitive transcription pathways relevant for fibroblast biology. When investigated, we highlight how the function of Ca^2+^-handling proteins changes during cardiac and pulmonary fibrosis. Many aspects of Ca^2+^ signaling remain unexplored in different types of cardiovascular fibroblasts in relation to pathologies, and a better understanding of Ca^2+^ signaling in fibroblasts will help to design targeted therapies against fibrosis.

## 1. Introduction

Fibroblasts represent a large group of heterogeneous, non-differentiated cells of mesenchymal origin that are ubiquitously expressed among organs, accounting for about one-third of the cell mass in our body [1]. Fibroblasts adopt several different phenotypes, which include quiescent (inactive) cells, pre-activated (proliferating) cells, or activated cells (myofibroblasts) [2,3]. Depending on their phenotype, fibroblasts appear in vitro as spindle-shaped (e.g., quiescent cells) or star-shaped cells (e.g., myofibroblasts) in culture. In vivo, they are largely part of fibrous connective tissues, and one important biological function is to secrete proteins that constitute the extracellular matrix (ECM). The secretion of ECM components is a hallmark of myofibroblasts, which often also express alpha smooth muscle actin (α-SMA), a contractile protein that promotes cell migration and contraction, and thus supports tissue repair. Both the secretion of ECM components, such as collagen, and the presence of α-SMA are frequently used as biomarkers for the myofibroblast phenotype [4]. Depending on the type of tissue, the cellular origins of fibroblasts vary, as fibroblasts can derive from various cells via a process termed epithelial-to-mesenchymal transition (EMT), as is the case for the majority of cardiac fibroblasts [5,6] or represent resident cells differentiated from local mesenchymal cell populations; for example, vascular fibroblasts in the adventitial layer [7,8]. Within organs, there is often a population of silent, resident fibroblasts, which start to proliferate and activate in response to physiological stimuli, such as cytokines or growth factors. The heterogeneity of fibroblast origins, as well as their ability to adopt multiple phenotypes, control important biological processes, such as wound healing and scar formation [9].

The activation of fibroblasts often occurs in response to tissue damage or inflammation [10], and while phenotype changes and migration of fibroblasts are necessary during organ development and tissue repair, chronic activation of fibroblasts in adult organs facilitates pathological tissue fibrosis implicated in organ dysfunction, such as heart [11], blood vessels [12], or lung [13]. Physiologically, following tissue repair, myofibroblasts undergo apoptosis in most tissues, but in the heart, it has been shown that cardiac myofibroblasts persist at the lesion site, such as infarct scars, which supports the maintenance and progression of cardiac fibrosis [14]. Therefore, to treat or delay fibrosis progression, it is important to acquire a complete picture of the underlying molecular signaling mechanisms in fibroblasts. In this context, many fundamental biological functions, such as secretion of vesicle content; proliferation, migration, and contraction of myofibroblasts; and gene expression depend on intracellular Ca^2+^ ions [15,16,17]. Therefore, analyzing the changes in fibroblast Ca^2+^ signaling that occur during diseases helps to better understand those cellular processes underlying pathological fibrosis. This review summarizes the current knowledge of signaling pathways leading to intracellular Ca^2+^ increase in fibroblasts. We will highlight how changes in Ca^2+^ signals may contribute to excessive fibrosis observed during pathologies of the cardiovascular system.

## 2. Receptor-Mediated Ca^2+^ Signaling via Phospholipase C (PLC)

**Figure 1:**
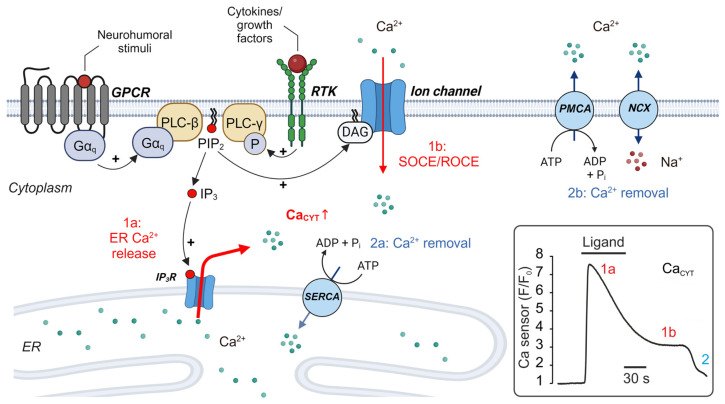
Regulation of cytoplasmic calcium (Ca_CYT_) in fibroblasts. Ca_CYT_ increases following stimulation of plasmalemmal receptors and activation of PLC, which triggers Ca^2+^ release from the ER (1a) and subsequent Ca^2+^ influx via Ca^2+^-permeable ion channels (1b). Ca^2+^ events are terminated by Ca^2+^-dependent closure of IP_3_R at high Ca_CYT_ levels and resting Ca^2+^ levels are re-established by Ca^2+^ transport processes that pump Ca^2+^ from the cytoplasm into the ER (2a) or extrude Ca^2+^ to the extracellular space (2b). Inset: simplified time course of intracellular Ca^2+^ changes in non-excitable cells induced by IP_3_R-mediated Ca^2+^ release and SOCE. Abbreviations: ADP: adenosine diphosphate. ATP: adenosine triphosphate. DAG: diacylglycerol. ER: endoplasmic reticulum. Gα_q_: G alpha q subunit of a heterotrimeric G protein (the βγ-dimer is not displayed for simplicity). GPCR: G protein-coupled receptor. IP_3_: inositol trisphosphate. PIP_2_: phosphatidylinositol bisphosphate. PLC: phospholipase C. NCX: Na^+^/Ca^2+^ exchanger. PMCA: plasmalemmal Ca^2+^-ATPase. SERCA: sarcoplasmic/endoplasmic Ca^2+^-ATPase. RTK: receptor tyrosine kinase. SOCE: store-operated Ca^2+^ entry. ROCE: receptor-operated Ca^2+^ entry. This figure was created in BioRender, Toronto, ON, Canada. Rinne, A. (2024) BioRender.com/x04y273 (accessed on 23 October 2024).

In most non-excitable cells such as fibroblasts, the release of Ca^2+^ from intracellular stores is initiated by the activation of inositol trisphosphate (IP_3_) receptors (IP_3_R) rather than ryanodine receptors (RyR) [18,19]. IP_3_ is generated following receptor-mediated stimulation of phospholipase C (PLC). Human fibroblasts express two PLC isoforms: PLCβ, which is activated by heterotrimeric G_q_ proteins released from activated G protein-coupled receptors (GPCR); and PLCγ, which is activated by phosphorylation via receptor tyrosine kinases (RTK, Figure 1 [20]. PLC cleaves the membrane phospholipid phosphatidylinositol bisphosphate (PIP_2_) into the messenger molecules IP_3_ and diacylglycerol (DAG), which activates protein kinase C (PKC) [21] and Ca^2+^-permeable ion channels, such as transient receptor potential (TRP) channels [22]. IP_3_ diffuses to ligand-gated Ca^2+^ release channels of the endoplasmic reticulum (ER), IP_3_R, which open and release Ca^2+^ from the ER into the cytoplasm (Ca^2+^ release, 1a in Figure 1). Ca^2+^ release is followed by Ca^2+^-influx from the extracellular space either via Ca^2+^ release-activated Ca^2+^ (CRAC) channels, the store-operated Ca^2+^ entry (SOCE) [23] or via Ca^2+^-permeable channels that bind DAG to open, such as some TRP channels (receptor-operated Ca^2+^ entry, ROCE, #1b in Figure 1) [24]. So far, receptor-mediated SOCE has been shown to regulate Ca_CYT_ in cardiac [25] and pulmonary fibroblasts [16], but not in vascular fibroblasts (see below). Both events, the initial Ca^2+^ release and the subsequent Ca^2+^ entry yield to a typical change in cytoplasmic Ca^2+^ (Ca_CYT_), the Ca^2+^ transient, which is depicted in the inset of Figure 1. The rise in Ca_CYT_, from ~100 nM at rest to ~1 µM at peak response, is recognized by Ca^2+^-sensing proteins, such as calmodulin (CaM), which integrate Ca^2+^ signals and cellular responses by controlling cellular effectors, including ion channels and enzymes [26].

In addition to IP_3_R, activation of ryanodine receptors (RyR) represents an alternative pathway for the release of Ca^2+^ from intracellular Ca^2+^ stores [17]. There are three RyR isoforms (RyR1 to RyR3), which are expressed in many different cell types to various expression levels, with RyR2 being the dominant isoform in cardiac myocytes. RyR2 is activated by binding Ca^2+^ ions to its cytoplasmic site following local increases in Ca_CYT,_ e.g., following Ca^2+^ influx via ion channels or single Ca^2+^ release events from the ER by adjacent IP_3_R or RyR, and inactivated at high concentrations of Ca_CYT_ at the end of release, similar to IP_3_R [18,27,28,29]. So far, there is no experimental evidence for a RyR2-mediated Ca^2+^ release mechanism in cardiac fibroblasts analogous to Ca^2+^-induced Ca^2+^ release (CICR) of cardiac myocytes [30]. For example, no mRNA transcripts encoding for one of the three RyR isoforms were detected in cardiac fibroblasts from rat or human, [31] and functional assays showed that application of caffeine (promoting RyR activation) did not evoke Ca^2+^ release from the ER [19]. Furthermore, intracellular Ca^2+^ signals in human cardiac fibroblasts were sensitive to the IP_3_R blocker 2-aminoethoxydiphenyl borate (2-APB), but not to ryanodine, which inhibits the RyR [19]. Moreover, L-type Ca^2+^ currents, which carry the Ca^2+^ influx that is necessary to trigger CICR, could not be recorded in human cardiac fibroblasts [25,31] (see also Section 4, “Ca^2+^-permeable ion channels” below). One study showed that RyR2 knockout mice displayed reduced cardiac fibrosis in atria in response to mechanical stretch. However, this effect was attributed to reduced paracrine myocyte-to-fibroblast signaling and not to a fibroblast-specific loss in RyR2 signaling [31]. On the other hand, there is some evidence for RyR-mediated signaling in pulmonary fibroblasts: TGF-β-evoked Ca^2+^ waves recorded in pulmonary fibroblasts were indeed sensitive to ryanodine, but the underlying RyR isoform was not specified [32]. To our best knowledge, there are no data on RyR3 expression or function in cardiac or pulmonary fibroblasts. Other intracellular Ca^2+^ release pathways such as RyR-like channels activated by cyclic ADP-ribose, sphingosine and a distinct Ca^2+^-release pathway activated by nicotinic acid adenine dinucleotide phosphate (NAADP) were identified in diseased cardiac myocytes [33] but have not been identified in fibroblasts. Thus, the potential role of RyR in fibroblast Ca^2+^ signaling is probably emerging but more experimental evidence is required to define such a pathway.

The large increase in Ca_CYT_ during release activates two mechanisms which lead to the termination of the cytoplasmic Ca^2+^ signal: high Ca_CYT_ concentration causes (i) IP_3_R to close, which terminates the release process [34,35] and (ii) activates Ca^2+^ transport mechanisms that re-establish resting Ca^2+^ levels by transporting Ca^2+^ back into the ER via the sarco-/endoplasmic Ca^2+^ ATPase (SERCA, #2a in Figure 1) [36], or by extruding Ca^2+^ from the cytoplasm by plasmalemmal Ca^2+^ ATPases (PMCA, #2b in Figure 1) [37]. A third Ca^2+^ extrusion mechanism in fibroblasts that affects Ca_CYT_ is the Na^+^/Ca^2+^ exchanger (NCX, 2b in Figure 1) [38,39,40]. Sometimes, termination is incomplete and the intracellular Ca^2+^ signal fluctuates and generates periodic Ca^2+^ oscillations due to the repetitive activation of IP_3_R [18]. Each distinct Ca^2+^ signal, short Ca^2+^ transients, longer-lasting Ca^2+^ waves, or periodic Ca^2+^ oscillations controls specific cellular responses [41].

Cardiovascular GPCRs that activate the canonical G_q_-PLCβ-IP_3_-Ca pathway in fibroblasts include AT_1_-R for angiotensin II (Ang II); purinergic P2YR for adenosine diphosphate (ADP), adenosine trisphosphate (ATP) and uridine triphosphate (UTP) [42]; B_2_R for bradykinin and ET_1_-R for endothelin-1 (ET-1) [43]; and the Ca^2+^-sensing receptor (CaSR) [44] and receptors for oxytocin [45]. The efficiency of this receptor–G_q_-PLC pathway can be enhanced by the co-activation of G_i/o_ proteins, as PLCβ can also bind the βγ-dimer released from the G_i/o_ protein simultaneously to G_q_ to facilitate PLC activation. In skin fibroblasts, the co-stimulation of G_q_- and G_i/o_ protein-coupled receptors resulted in enhanced Ca^2+^ release from intracellular stores as compared to G_q_ protein signaling alone [46]. In cardiac fibroblasts, Ang II has been established as a key signaling molecule to drive fibrosis. Paracrine Ang II activates SOCE and increases Ca_CYT_ in cardiac fibroblasts [47], which stimulates their transition to myofibroblasts [48]. This effect is even further amplified in fibrotic cardiac tissue, where myofibroblasts secrete Ang II and upregulate their own AT_1_R expression, which generates an autocrine signaling pathway leading to continuous fibroblast activation [49]. Purinergic signaling seems to control the activation of cardiac fibroblasts in an agonist-dependent fashion. Stimulation of P2Y_2_R (a G_q_- and G_12/13_-coupled receptor) with ATP promoted cardiac fibroblast proliferation [50], whereas the activation level of A_2B_ adenosine receptors (A_2B_AR, a G_q_- and G_s_- coupled receptor) has been implicated in regulating basal collagen synthesis rates in cardiac fibroblasts [51]. Other GPCRs which are upregulated during fibrosis in disease models for heart-, lung-, liver-, and kidney fibrosis include GPR176, α_2_ adrenergic receptor (α_2_-AR), P2Y_13_, B_1_R for bradykinin, 5-HT receptors, as well as chemokine and cytokine receptors [52]. Interestingly, a regulator of GPCR signaling, the G protein-coupled kinase 5 (GRK5), was upregulated in a myocardial infarction model. Despite its primary function of shaping and terminating GPCR signaling by receptor phosphorylation, it served as a co-factor for fibroblast transcription by translocating into the nucleus in a Ca^2+^-dependent fashion [53] (see Section 6 below).

Another pathway for Ca^2+^ release relies on the activation of receptor tyrosine kinases (RTK), which stimulate PLCγ isoforms at the plasma membrane and liberate Ca^2+^ from intracellular Ca^2+^ stores via IP_3_R [54]. In fibroblasts, the RTK agonists epidermal growth factor (EGF), vascular epidermal growth factor (VEGF), together with the serine/threonine kinase receptor agonist transforming growth factor beta (TGF-β), activates either PLCγ-IP_3_-Ca^2+^ signaling (Ca^2+^ release) and/or Ca^2+^ influx from the extracellular space [55,56]. In some cases, PLCγ activates ROCE directly via DAG (#1b in Figure 1), presumably without contribution of previous ER Ca^2+^ release [57]. Little is known about whether the expression and activity levels of RTK are altered in diseased cardiovascular fibroblasts. However, RTK have been implicated in regulating proliferation and the escape from apoptosis in cancer-associated fibroblasts [58]. Thus, more studies are required to define those changes in receptor signaling that promote fibrosis.

Both signaling pathways, activation of GPCR and RTK, have been implicated in vascular fibrosis. Progressive fibrosis occurs in the adventitia, the outer layer of vessels and causes stiffening of the vessel wall, which changes hemodynamic properties, reduces tissue perfusion rates and may lead to tissue damage [59]. Remodeling of the vessel wall is accompanied by a change in the composition of the ECM caused by fibroblast activation, which increases the secretion rates of collagen and fibronectin [60]. Among the many signaling molecules that cause fibroblast activation and collagen synthesis are Ang II [61] and ET-1 [62], both of which are excessively released during hypertension or other cardiovascular diseases (CVD) [63]. And while Ang II-induced vascular fibrosis has been linked to NADPH-Oxidase 2 (NOX2) activity [64], there is, to our best knowledge, no detailed study on Ang II-induced Ca^2+^ signals in vascular fibroblasts. Another signaling molecule that has been implicated in vascular fibrosis is TGF-β, which acts via the serine/threonine kinase receptor TGFβR and which also induces secretion of vascular ECM compounds [65,66]. TGF-β itself is released by Ang II-activated smooth muscle cells [67], indicating that both Ang II and TGF-β may act on vascular fibroblasts simultaneously. In summary, functional studies addressing vascular fibroblast Ca^2+^ signaling are sparse. Taking into consideration the fibroblast heterogeneity and that less than 20% of genes expressed in fibroblast overlapped between fibroblasts isolated from heart, skeletal muscle, intestine or bladder [68], more studies are required to describe the particularities of the molecular signaling pathways underlying Ca^2+^ signaling in vascular fibroblasts.

Another class of fibroblast-activating substances are inflammatory agents released by mast cells or damaged tissues to induce repair and scar formation. For example, during cardiac injuries, CC chemokine-receptor 2 positive (CC2+) macrophages release interleukins (IL) and tumor necrosis factor α (TNF-α), both of which promote cardiac fibroblast activation [69]. Furthermore, resident mast cells infiltrate lesion sites and recruit neutrophiles and there is a clear correlation between the number of infiltrated mast cells (mast cell density) and collagen production by activated cardiac fibroblasts, eventually resulting in fibrosis and heart failure [70,71]. Macrophage activation contributes to sustained tissue inflammation and fibroblast activation in the heart [72] and, in turn, messenger molecules released by activated fibroblasts, such as colony-stimulating factor 1 (CS-F1) may result in additional recruitment and activation of macrophages, which further facilitates inflammatory tissue responses. Therefore, the interplay between inflammation and activation of fibroblasts may create a vicious circle to facilitate organ fibrosis [73,74,75].

## 3. Receptor-Mediated Regulation of Nuclear Ca^2+^ (Ca_NUC_)

**Figure 2:**
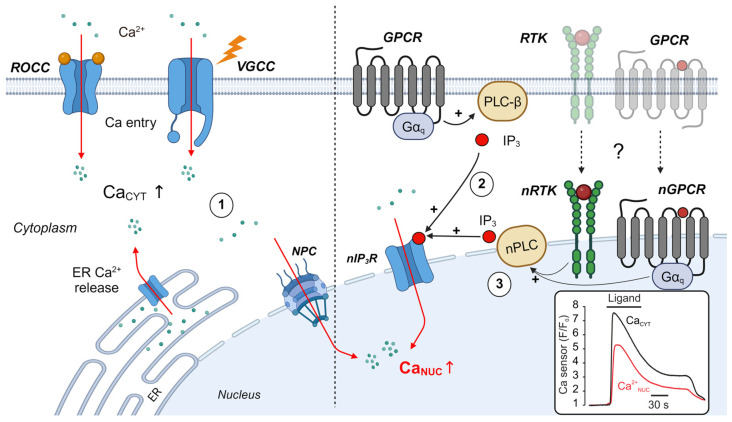
Regulation of nucleoplasmic calcium (Ca_NUC_) in fibroblasts. Ca_NUC_ increases by one of three mechanisms: (1) A large cytoplasmic elevation of Ca^2+^ causes an increase in Ca_NUC_ by diffusion of Ca^2+^ into the nucleus via the NPC. (2) Activation of plasmalemmal PLC generates IP_3_, which diffuses to nuclear IP_3_R (nIP_3_R) and generates a local Ca^2+^ signal. (3) Direct stimulation of nuclear PLC (nPLC) by nuclear receptors generates a local IP_3_-Ca^2+^ signal. Following their activation at the plasma membrane, some receptors may translocate to the nuclear envelope in an active conformation to generate local IP_3_ signaling. So far, it is unknown whether the nIP_3_R, translocated receptors and nPLC are localized at the outer or the inner nuclear membrane. Abbreviations: NPC: nuclear pore complex. nGPCR: nuclear GPCR. nRTK: nuclear RTK. ROCC: receptor-operated cation channel. VGCC: voltage-gated Ca^2+^ channel. Inset: representative comparison of the time courses of Ca_CYT_ and Ca_NUC_ in non-excitable cells caused by Ca^2+^ diffusion into the nucleus. Black trace: simplified representation of Ca_CYT_ (from Figure 1) as assessed with a cytoplasmic Ca^2+^ biosensor. Red trace: rapid diffusion of cytoplasmic Ca^2+^ into the nucleus in cells exposed to the same stimulus as assessed with a Ca^2+^ biosensor localized to the cell nucleus [78]. This figure was created in BioRender, Toronto, ON, Canada. Rinne, A. (2024) BioRender.com/x04y273 (accessed on 23 October 2024).

The nuclear Ca^2+^ content (Ca_NUC_) regulates many important biological functions, including gene transcription and cell cycle progression [76]. A rise in Ca_NUC_ is regulated by at least three different mechanisms (Figure 2): (i) Ca^2+^ can rapidly diffuse from the cytosol into the nucleus through the nuclear pore complex (NPC) and any rise in Ca_CYT_ will therefore affect Ca_NUC_ (#1 in Figure 2) [77]. The inset in Figure 2 shows this principle: the two traces display representative changes in Ca_CYT_ (black trace) or Ca_NUC_ (red trace) in response to GPCR stimulation, as assessed with genetically encoded Ca^2+^ indicators targeted to the cytoplasm or to the nucleus, respectively [78]. It is evident that when cells generate a large cytoplasmic Ca^2+^ signal, diffusion of Ca^2+^ into the nucleus occurs fast and Ca_NUC_ is simply dominated by Ca_CYT_. (ii) There is, however, some evidence for local Ca^2+^ signaling at the nuclear envelope, which could be, at least theoretically, independent from cytoplasmic Ca^2+^ signaling events. In the first scenario, IP_3_ is generated by plasmalemmal PLC stimulation and diffuses to IP_3_R embedded in the nuclear envelope, which generates a local Ca^2+^ signal (#2 in Figure 2) [79]. Because the nuclear envelope is an extension of ER membranes, it may serve as a Ca^2+^ store for the nucleus and activation of nuclear IP_3_R controls Ca_NUC_ [80,81]. While the concept of IP_3_ diffusion has been initially described for cardiac myocytes [82], it is unclear whether IP_3_ generated at the plasmalemma can affect Ca_NUC_ without generating a large cytoplasmic Ca^2+^ signal via ER-Ca^2+^ release in fibroblasts [83]. (iii) Alternatively, a local PLC-IP_3_-Ca^2+^ signal could be generated by receptors localized to the nuclear envelope. For example, α_1_AR, B_2_R, ET_1_R and AT_1_R, all of which activate PLC, contain nuclear localization sequences and have been identified to localize to the nuclear envelope of cardiac fibroblasts, cardiac myocytes and endothelial cells. Importantly, the nuclear envelope also contains functional PLC isoforms [84]. It has been demonstrated that the activation of those nuclear GPCR caused local increases in Ca_NUC_, which has been implicated in controlling gene transcription [85,86,87,88,89,90,91].

While the functionality of nuclear localized GPCR has been demonstrated using intracellular uncaging of synthetic receptor ligands [92], it remains to be demonstrated how endogenous ligands may reach intracellular receptors under physiological signaling conditions. Three different mechanisms have been proposed for the stimulation of nuclear receptors in fibroblasts and other cell types: (i) following their synthesis, some intracrine ligands may be retained in the cytoplasm of the cell and could be transported to the nuclear envelope [93,94,95]; (ii) ligands may be transported from the extracellular space into the cytoplasm by cellular uptake mechanisms [96]; and (iii) activated receptors may translocate from the plasma membrane to the nuclear envelope via internalization (#3 in Figure 2) [97]. Indeed, some GPCR internalize with their agonists bound, retain their signaling activity in endosomes [98], and may appear at the nuclear envelope following their activation at the plasma membrane [99]. This mechanism of receptor translocation appears attractive, since it may generate long-lasting nuclear Ca^2+^ signals that persist even when cytoplasmic Ca^2+^ elevations have faded away. Receptor translocation to the nucleus has been demonstrated for receptors for EGF and TGF-ß, which are relevant for fibroblast signaling. Following their stimulation at the plasma membrane, activated receptor molecules translocate to the cell nucleus, where they control gene transcription via Ca^2+^-dependent and Ca^2+^-independent mechanisms [100,101,102]. In line with the proposed mechanism, for some, RTK activation of nuclear PLCγ appeared to be independent from plasmalemmal PLC activation [103]. In addition, following stimulation of fibroblasts with insulin-like growth factor I (IGF-1), local production of DAG and PKC activation was detected at the nuclear envelope, highlighting the existence of nuclear PLCγ signaling [104,105]. Moreover, Ca_NUC_ has been implicated in regulating gene transcription via Ca^2+^-sensitive transcription factors independently from Ca_CYT_ [83,106]. Irrespective of the activating mechanism, such “intracrine” or “compartmentalized” receptor signaling events represent a novel signaling concept that may be implicated in multiple cardiovascular diseases, including fibroblast activation and fibrosis [95].

## 4. Ca^2+^-Permeable Ion Channels

Ca^2+^ influx from the extracellular space regulates many biological functions of fibroblasts, including cell proliferation and myofibroblast migration. Fibroblasts express voltage-gated Ca^2+^ channels and receptor-activated ion channels underlying SOCE and ROCE (Figure 1). The membrane potential (V_M_) of cardiac fibroblasts reaches values between −40 mV and −15 mV at rest [107,108]. However, during cell proliferation, membrane potential values of fibroblasts may reach even more depolarized values up to −10 mV [109,110], while during myofibroblast activation the membrane potential can hyperpolarize to −55 mV [111], suggesting a correlation of V_M_ with the differentiation state of fibroblasts. Human fibroblasts express various K^+^ channels that regulate V_M_, including voltage-gated (K_V_) channels, inwardly rectifying K^+^ (K_ir_) channels and Ca^2+^-activated K^+^ channels (K_Ca_), among others [112,113,114]. Changes in fibroblast K^+^ channels expressions have been implicated in fibroblast-related pathologies of the cardiovascular system, affecting the resting membrane potential in fibroblasts [115]. Changes in V_M_ may not only alter the activity of voltage-gated Ca^2+^ channels (VGCC), but also affect the electrochemical driving force for Ca^2+^ entry via non-voltage-gated Ca^2+^ channels [17,115], which are discussed below.

**Voltage-gated Ca^2+^ channels.** There are two main VGCC in human fibroblasts: low voltage-activated (T-type) Ca^2+^ channels and high voltage-activated (L-type) Ca^2+^ channels, both of which are expressed in human fibroblasts [116,117]. Ca^2+^-influx via VGCC has been shown to generate Ca^2+^ oscillations in lung fibroblasts, which have been implicated in regulating fibroblast proliferation and secretion of ECM components, suggesting that VGCC may represent potential drug targets to prevent lung fibrosis [117]. In contrast, the role of VGCC is less well defined for cardiac fibroblasts. Whereas mRNA transcripts encoding for both VGCC types were detected [19,118], most studies highlight that there is a lack of functional expression of L-type calcium channels in human cardiac fibroblasts [19,119], suggesting that alternative Ca^2+^ influx pathways were more relevant for Ca^2+^ signaling of cardiac fibroblasts than VGCC. There is, however, experimental evidence that Ca^2+^ channel expression may change with fibroblast activation. One study found that TGF-ß stimulation of cardiac fibroblasts resulted in activation of SMAD transcription factors, which transduce TGF receptor activation into cellular responses, and was sensitive to a combined pharmacological inhibition of T-type and L-type channels [120]. Furthermore, currents of both, T-type and L-type channels, were recorded from human activated myofibroblasts, suggesting that ion channel expression may be upregulated during fibroblast stimulation in vitro or during their pathologic activation in vivo [118]. Therefore, the role of VGCC in cardiac fibroblasts requires more thorough electrophysiological investigation with respect to fibroblast phenotypes.

**SOCE and ROCE.** SOCE represents the major Ca^2+^ signaling pathway of all non-excitable cells [121,122]. It is activated by ER Ca^2+^ release when functional Orai channels, the ion channels underlying the Ca^2+^ release-activated Ca^2+^ current (I_CRAC_), are formed at the plasma membrane. ER Ca^2+^ release is sensed by luminal Ca^2+^ sensors, stromal interaction molecules 1 (STIM1) proteins, which are activated at low ER Ca^2+^ concentrations and which mediate the formation of functional, hexameric Orai channels by direct coupling [123,124,125,126]. Many neurohumoral factors or cytokines activate the PLC-IP_3_-SOCE pathway in fibroblasts (Figure 1). Ca^2+^ entry via SOCE causes the plateau phase of the Ca^2+^ transient (# 1b in Figure 1), the capacitative Ca^2+^ entry [124], which has been shown to activate Ca^2+^-sensitive transcription factors, such as nuclear factor of activated T cells (NFAT) in most non-excitable cells [127,128]. NFAT controls cell proliferation by regulating gene expression of cyclins, which, in turn, control transitions between cell cycle phases [129,130]. Another important Ca^2+^ signal stems from periodic activation of IP_3_R and SOCE to generate Ca^2+^ oscillations, which regulate phase transitions and the synthesis of DNA during the S-phase of the cell cycle [131]. In fibroblasts, SOCE-mediated Ca^2+^ oscillations are key signals for the induction of cell proliferation [19]. Chronic activation of NFAT signaling following GPCR activation has been implicated in pathological fibroblast activation during cardiac- and lung fibrosis [23,132,133,134]. As an underlying Ca^2+^ source, an increase in Ca^2+^-influx via SOCE in human cardiac fibroblasts has been identified for age-related fibrosis [135] and fibrosis during heart failure [136], suggesting that SOCE indeed drives pathology-related remodeling of fibroblasts.

ROCE represents a Ca^2+^ influx pathway, which is defined here as Ca^2+^ entry via ligand-gated channels. In many non-excitable cells, this includes purinergic P2X receptors, which are non-selective cation channels gated by ATP and other nucleotides [137]. ATP binding generates a direct Ca^2+^ influx from the extracellular space [138] and, as opposed to the typical Ca^2+^ transient elicited by P2YR, P2X activation generates a rather long-lasting Ca^2+^ signal with low amplitude and corresponding membrane depolarization in fibroblasts. P2XR activation mediated inflammation and fibrosis of the lungs and fibrosis of the heart (P2Y_6_ and P2X_7_) [139,140,141,142,143]. In cells that express several P2X and P2Y receptors, the delineation of cellular responses specifically mediated by P2X receptors may prove difficult and relies on the availability of specific P2X blockers or specific allosteric modulators as well as the generation of knockout mice [144,145]. Furthermore, activation of P2XR in neutrophils and macrophages (e.g., P2X_7_R) by ATP induces strong inflammatory responses [146], and inflammatory mediators, such as interleukins and cytokines, which are strong activators of fibroblasts. Thus, P2X signaling may induce fibrosis via both, Ca^2+^-dependent and Ca^2+^-independent pathways. Targeting P2X_7_R has been suggested as a pharmacological strategy to interfere with fibrosis in the cardiovascular system and other organs [147,148].

**TRP channels.** TRP channels are a large family of non-specific, Ca^2+^-permeable cation channels. They are often activated by a combination of biochemical and physical stimuli, including extracellular ligands, temperature, and intracellular signaling molecules, such as DAG (for review see: [149]). There is a large body of evidence that TRP channel activation generates Ca^2+^ signals that control fibroblast proliferation and their phenotype transitions, as reviewed in [25]. Members of the TRPC family (e.g., TRPC3 and TRPC6) are regulated by DAG following receptor/PLC activation (Figure 1) and have been demonstrated to activate NFAT signaling in a Ca^2+^-dependent fashion in the heart [150]. In particular, TRPC3 has been identified as a key Ca^2+^ influx pathway that activates the NFAT transcription pathway and induces hypertrophy and fibrosis in the heart [151]. Pharmacological inhibition of TRPC3 reduced cardiac fibrosis in a mouse model for pressure-induced cardiac hypertrophy [152], an effect that was attributed to the reduced Ca^2+^-dependent activity of NOX2 [153]. It is noteworthy that specific inhibition of TRPC3 prevented Ca^2+^-dependent activation of NFAT [154], which precedes cardiac hypertrophy and fibrosis, suggesting TRPC3 channels as novel drug targets to prevent cardiac fibrosis in the human heart.

In concert with the activation of TGF-ß receptors, which in turn, regulate TRP channel expression, the activity of those channels induces the cardiac fibroblast-to-myofibroblast differentiation [155], a key process for the onset of severe cardiac fibrosis [156]. Activation of cardiac fibroblasts following stimulation with Ang II or TGF-β was mediated by TRPC6, which activated the p38-MAPK pathway in a Ca^2+^-dependent fashion [157]. In the lungs, several members of the TRP channel family are involved in the onset of idiopathic pulmonary fibrosis. For example, activation of TRPV4, a channel that is activated by osmotic changes and modulated by PKC-phosphorylation and alterations in PIP_2_ plasma membrane content following PLC activation [158,159], causes Ca^2+^ influx and strong induction of lung fibrosis [159,160]. Other examples include members of the TRPC- or TRPV family, which either function as homomeric or heteromeric channels, mediating Ca^2+^ influx in response to PLC stimulation and binding of DAG. It is speculated that Ca^2+^ entry via heteromeric TRP channels may be amplified during fibrotic pathologies [161]. Because the activation of TRP channels is multimodal and not restricted to one activating stimulus alone, it often proves to be difficult to delineate the precise signaling mechanisms that lead to TRP-induced fibrosis in a particular tissue. Nevertheless, specific pharmacological modulation of individual TRP channel species or channel assemblies may provide a promising strategy to prevent pulmonary fibrosis or other fibrotic diseases [162,163].

## 5. Removal of Ca^2+^: Ca^2+^ Pumps and Transporters

Whereas most research focuses on Ca^2+^-handling proteins that increase Ca_CYT_, Ca^2+^ removal and uptake mechanisms play a similar important function for Ca^2+^ signaling, since they shape the duration of Ca^2+^ signals and terminate Ca^2+^ events. There are three major molecular mechanisms to clear the cytoplasm from excessive Ca^2+^, which re-establishes resting Ca^2+^ levels in fibroblasts: first, Ca^2+^ is pumped back into the ER via SERCA, which primes the cell for the next Ca^2+^ release event (2a in Figure 1). Second, Ca^2+^ ions are extruded to the extracellular space via NCX, which uses the Na^+^ gradient that has been established by the Na^+^/K^+^-ATPase across the plasma membrane (2b in Figure 1). Analogue to diseased cardiomyocytes, NCX has been implicated in generating pathologic Ca^2+^ signals in cardiac myofibroblasts by operating in reversed mode [39,40]. The third mechanism is Ca^2+^ extrusion via plasmalemmal Ca^2+^ ATPases (PMCA, #2b in Figure 1) [19]. Whereas the focus of interest in Ca^2+^ extrusion mechanisms in cardiac myocytes lies mainly on the first two mechanisms [30], PMCA are highly relevant for Ca^2+^ extrusion in fibroblasts. There are four isoforms of PMCA (PMCA 1-4), which are ubiquitously expressed among tissues [164], including fibroblasts [19]. PMCA are probably the main Ca^2+^ extrusion pathway of non-excitable cells and activation of PMCA regulates the shape and duration of an intracellular Ca^2+^ transient [165], as well as total Ca_CYT_. In cardiac fibroblasts, changes in PMCA activity alters Ca^2+^ signaling, which translated into a change in their biological function: a study that had used fibroblasts from PMCA4 knockout mice could demonstrate that a lack of PMCA4 prevented the pressure overload-induced hypertrophy of cardiac myocytes in a paracrine fibroblast-to-myocyte signaling fashion by a mechanism that involved increased secretion of secreted frizzled related protein 2 (sFRP2) by fibroblasts. Consequently, pharmacological inhibition of PMCA4, which is less relevant for cardiac myocyte Ca^2+^ signaling, may present a promising strategy to prevent cardiac hypertrophy [166].

## 6. Ca^2+^-Dependent Transcription Processes in Fibroblasts

**Figure 3:**
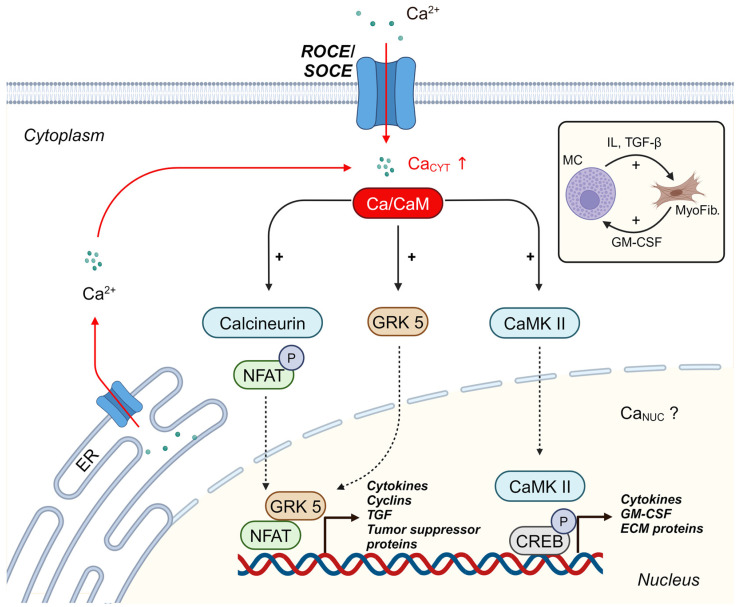
Ca^2+^-dependent transcription processes in fibroblasts. In the first step, an increase in Ca_CYT_ activates the Ca^2+^ sensor protein CaM. The Ca^2+^/CaM complex activates three effectors in the cytoplasm: (1) Activation of the phosphatase calcineurin controls NFAT transcription factors. Inactive NFAT is highly phosphorylated and resides in the cytoplasm. Calcineurin dephosphorylates NFAT, which causes a translocation of the active transcription factor into the cell nucleus to induce gene transcription. In cardiac fibroblasts, Ca^2+^/CaM also induces the translocation of GRK 5 to the cell nucleus, where it serves as a co-factor for NFAT. (2) In cardiac fibroblasts, Ca^2+^/CaM also induces the translocation of GRK 5 to the cell nucleus, where it serves as a transcriptional co-factor for NFAT. (3) CaM activates the kinase CaMK II. Activated CaMK II is transported into the nucleus, where it activates CREB transcription factors by direct phosphorylation. Inset: some gene products of fibroblasts stimulate mast cells (MC). MC, in turn, release stimulators of fibroblasts, which creates a vicious circle during tissue inflammation. Abbreviations: CaM: calmodulin. CaMKII: Ca^2+^/CaM-dependent kinase II. CREB: cAMP response element binding protein. GM-CSF: granulocyte–macrophage colony-stimulating factor. GRK: G protein-coupled receptor kinase. IL: interleukin. MyoFib.: myofibroblast. NFAT: nuclear factor of activated T cells. TGF: transforming growth factor. This figure was created in BioRender, Toronto, ON, Canada. Rinne, A. (2024) BioRender.com/x04y273 (accessed on 23 October 2024).

All biological functions that are regulated by Ca^2+^ ions in fibroblasts, such as cell proliferation, cell migration, and secretion of bioactive compounds, require changes in gene transcription. Changes in Ca_CYT_ are sensed by intracellular Ca^2+^ sensor proteins, which integrate Ca^2+^ signaling with cellular responses. The Ca^2+^ sensor calmodulin (CaM), which binds four Ca^2+^ ions at two EF-motifs, is ubiquitously expressed in many cell types, including cardiac fibroblasts [167]. Following Ca^2+^ binding, the α-helical protein undergoes a conformational change that increases its affinity for effector proteins [168]. For example, stimulation of cardiac fibroblasts with Ang II generates a Ca^2+^ signal that activates CaM and induces the fibroblast-to-myofibroblast phenotype transition [169]. In its active conformation, cardiovascular CaM regulates numerous cellular targets, which are reviewed in detail in reference [170]. Among them are Ca^2+^/CaM-dependent kinase II (CaMK II) and the phosphatase calcineurin (CaN). Both enzymes are key regulators of transcriptional processes in the cardiovascular system. In the following section, we would like to briefly describe the activation of two Ca^2+^/CaM-regulated transcription factors (Figure 3): (i) activation of NFAT by CaN and (ii) activation of cAMP response element binding protein (CREB) by CaMK II.

**Activation of NFAT.** Cytoplasmic CaM activates the phosphatase calcineurin A (Figure 3), which regulates NFAT transcription factors by dephosphorylation. At rest, inactive NFAT is phosphorylated and resides in the cytoplasm. Dephosphorylation activates the transcription factor and induces its translocation from the cytosol to the nucleus, where it initiates gene transcription in conjunction with other cofactors [171]. Activation of NFAT is an important step in regulating cell proliferation. NFAT controls the gene transcription of regulatory proteins of the cell cycle, including cyclins, cyclin-dependent kinases and tumor suppressor genes [130]. For example, stimulation of Ca^2+^/CaN/NFAT pathway via α_1_-ARs induces proliferation of cardiac fibroblasts, linking a neurohumoral factor that is released in response to hypertension with the induction of organ fibrosis [172]. In fibroblasts derived from a heart of an animal model for cardiac infarction, GRK5 was activated in response to stimulation of the fibroblasts with Ang II. Surprisingly, following activation of Ca^2+^/CaM, GRK5 translocated from the cytoplasm to the cell nucleus where it enhanced the transcriptional activity of NFAT [53]. Another study related inflammatory ER stress to fibrosis of the lungs. The authors demonstrated that during the inflammatory response, synthesis of collagen was controlled by NFAT transcription factors in pulmonary fibroblasts in a Ca^2+^-dependent fashion following their exposure to TGF-β [173]. Thus, Ca^2+^-dependent activation of NFAT seems to be one important transcriptional pathway implicated in fibrosis.

**Activation of CREB.** A second target of cytoplasmic CaM is CaMK II (Figure 3). Activated CaMK II is imported into the nucleus, where it regulates several different transcription factors. CREB transcription factors are activated by CaMK II via direct phosphorylation of nuclear CREB [174]. In addition to its classical activation by β adrenergic receptors (β-ARs) via cAMP, in cardiac fibroblasts CREB has been shown to be activated via multiple alternative pathways: Ang II activates the PLC-IP_3_-Ca^2+^ pathway and CREB via CamK II-dependent phosphorylation (Figure 3). In a second pathway, CREB is activated independent of Ca^2+^ by the Ras/p38MAPK, which induces fibrosis following upregulation of the protein periostin. Finally, stimulation of β_2_-AR results in phosphorylation and activation of CREB via the cAMP-dependent, but Ca^2+^-independent protein kinase A (PKA) signaling pathway [175,176]. Activation of CREB has been implicated in promoting proliferation by regulating the expression of cyclins [177] as well as in facilitating myofibroblast activation and ECM protein deposition during cardiac fibrosis [178]. In pulmonary fibroblasts, stimulation of the TNF-α pathway activated CREB and enhanced the secretion of granulocyte–macrophage colony-stimulating factor (GM-CSF), which in turn, activated the inflammatory response of macrophages. GM-CSF causes further inflammation of the lungs, which may result in activation of even more quiescent fibroblasts in lung tissue [179]. In this context, it is worth noting that CREB activation frequently occurs via RTK receptors for inflammatory substances via multiple signaling pathways in a Ca^2+^-independent fashion [180].

## 7. Future Directions and Conclusions

The studies presented above represent a focus on the major Ca^2+^ signaling pathways in fibroblasts. While different Ca^2+^ signaling pathways, such as intracellular Ca^2+^ release or Ca^2+^ influx via receptor-operated and voltage-gated Ca^2+^ channels contribute to fibroblast biology, it is worth noting that Ca^2+^-independent pathways are also important for fibroblast biology; for example, the process of cell migration certainly involves Ca^2+^ ions, but cell movement is also fine-tuned by other cellular signaling aspects, such as phosphorylation status, oxidation or other posttranslational modifications of signaling proteins, as well as Na^+^- and K^+^ ion homeostasis, osmosis, or pH gradients (reviewed in: [181]). Therefore, robust fibroblast activation may involve the simultaneous activation of Ca^2+^-dependent and Ca^2+^-independent signaling pathways. The characterization of distinct Ca^2+^ handling proteins is also hampered by technical difficulties: quantifying fibroblast-to-myofibroblast phenotype changes with respect to cellular signaling, e.g., the up- or downregulation of Ca^2+^-handling proteins, depending on cell differentiation, requires good control over fibroblast phenotypes in culture. This appears difficult for two reasons: first, there are many more fibroblast phenotypes than just the “resting” and “activated myofibroblast”, depending on the tissue origin (reviewed in: [182]), an aspect that is important when analyzing primary fibroblasts. Second, there is some caution that cultured cardiac fibroblasts will already represent myofibroblasts, i.e., some standard 2D-culture conditions may induce fibroblast activation, per se [183]. Another aspect is that the number of fibroblasts obtained from primary tissue is usually low and those cells require several rounds of multiplication during numerous cell culture passages to reach the number of cells required for experiments and further passaging. Several studies have shown that the expression levels of ion channels seem to vary with increasing numbers of passaging and time in culture in primary fibroblasts (reviewed in: [184]). Therefore, it must be established how in vitro results reflect pathophysiological alterations of ion channels in vivo.

Furthermore, there is a strong interaction of fibroblast activity with the inflammasome in the sense that active immune cells secrete activators of fibroblasts, and vice versa [185]. Moreover, some fibroblasts directly interact with surrounding cells in vivo. This is highly relevant for cardiac fibroblasts, which alter the electric and metabolic properties of adjacent myocytes by direct electric coupling, as well as by paracrine and juxtracrine signaling (reviewed in: [186]). Thus, the phenotypes and signaling properties of primary fibroblasts may differ with prolonged time in culture, as the influence of inflammatory substances may fade when those cells are taken into culture for further experimental studies. Those aspects may render it difficult to compare different fibroblast studies, and to identify new drug targets to prevent excessive fibrosis.

A rather novel and unexplored aspect of fibroblast signaling is the potential dependence of Ca^2+^ signaling on pH. Tissue inflammation is characterized by extracellular acidosis and many membrane proteins are sensitive to protons. A newly discovered group of proton-sensitive membrane proteins are proton-sensing GPCRs, which have been characterized in many cell types, including fibroblasts and cardiomyocytes [187,188]. Those receptors contain extracellular proton-binding domains, show activity in a pH range between pH = 6.2 and pH = 7.4 and are stimulated by an increase in proton concentration [189]. The receptors can couple to more than one G protein species, but some receptors, such as GPR4, signal predominantly via the G_q_-PLC-IP_3_-Ca^2+^ pathway [188,190]. The stimulation of GPR4 may result in increased Ca^2+^ signaling during tissue acidosis in endothelial cells, but its functional role in cardiac fibroblasts is unknown. A second mechanism that links Ca^2+^ signaling to intracellular pH has been initially discovered in human neutrophils. During the unspecific cellular immune response, neutrophils produce reactive oxygen species in a biochemical process that involves activation of NOX. A byproduct of NOX activity are protons, which rapidly accumulate in the cytosol, and excessive acidification is prevented by compensatory activation of voltage-gated proton channels, yielding to proton efflux (reviewed in detail in [191]). In experiments using proton channel-deficient neutrophils, activation of the oxidase resulted in robust cytosolic acidification, which caused an unexpected reduction in Ca^2+^ influx via SOCE [192]. And while the molecular mechanism for this phenomenon has not been explored, those experiments demonstrated that a fall in intracellular pH can affect Ca^2+^ signaling. Human cardiac fibroblasts express proton channels [193] and NOX2 is often active in stimulated fibroblasts [153]. In such a scenario, the combination of NOX2 activation and reduced proton efflux during extracellular acidification could yield intracellular acidification in fibroblasts. It would be very interesting to investigate in future studies whether extracellular acidification will alter cardiac fibroblast Ca^2+^ signaling by either of those mechanisms.

Despite being regarded as structural cells, the previous decades of research that focused on fibroblasts changed the old concept about the physiological role of those cells and their contribution to pathology. Because of their heterogeneity and cell plasticity, fibroblasts may display tissue-specific responses in pathology. Thus, more investigations are required to describe molecular mechanisms underlying pathological responses in heart-, lung-, and vascular fibroblasts.
